# General Medicine and Therapeutics

**Published:** 1893-06

**Authors:** 


### *SELECTIONS AND ABSTRACTS.*

---

### GENERAL MEDICINE AND THERAPENTICS.

---

EDITED BY DR. LUTHER B. GRANDY.

---

### TYPHOID FEVER IN CHILDREN.

Dr. Earl (Arch. of Pedriat.) gives the following as nutritious, aiding digestion, and a mild intestinal antiseptic:

| | | |
|---|---|---|
| ℞. Pepsin cordial, | | ℥ i. |
| Syr. hypophos. comp., | | ʒ iv. |
| Acid. sulphur. arom., | | ʒ i. |
| Aquæ menth. piper., | q. s. ad | ℥ ii. |

M. d. Sig.—One teaspoonful, well diluted, four times daily. —*Ex.*

---

### FEEDING IN FEVERS.

Peabody (*Medical Record*, November 26, 1892) believes that in all kinds of illness, and especially in fevers, attention must be paid to the appetite and desire of the patients. If a patient is really hungry solid food of a properly selected kind, and in judicious quantities, will rarely disagree with him. He believes there is less danger in doing harm to an ulcerated ileum in typhoid fever by giving finely divided egg, beef or chop than by giving milk. He habitually gives his patients with typhoid, who are hungry, such food. He believes it is a mistake to withhold solid food merely because a patient has fever, and that it is incorrect to regard milk as a fluid food. Milk will always remain the most serviceable general food in disease, but where it fails to nourish the patient, where it is not well borne, where it cannot be taken for any reason, it is well to remember that efficient adjuncts and substitutes are within reach.—*Univ. Med. Mag.*

### TREATMENT OF CROUP.

Dr. N. S. Davis says all the indications for treatment in crop, in the mild or superficial form of the disease, can be filled by the administration of:

℞. Syr. scillæ co., ʒ iss.

Syr. ipecacuan, ʒ ix.

Tr. op. camph., ℥ ij.

M. Sig.—Half teaspoonful every three or four hours.—*Medical Record.*

---

### MINERAL WATERS.

The following is an abstract of a paper on "Artificial and Natural Mineral Waters," read by Prof. O. Liebreich at the Balneological Congress recently held in Berlin. He began by asking: Is chemistry sufficiently advanced yet to produce artificial mineral water equal in all respects to the natural water? The answer cannot be in the affirmative. Some substances, such as alizarine, indigo, urea, etc., can be produced synthetically, the artificial product being in every respect identical with the natural one. But in the case of mixtures such as mineral waters are, synthesis is a much more difficult matter. The analysis of each of the first mentioned substances gives exactly 100 per cent., whilst in the case of artificial mineral waters the analyses, even of the most renowned analysts, fall short of 100 per cent., thus leaving a remainder the nature of which is absolutely unknown.

Many mineral waters on evaporation leave an organic residuum which goes by the name of glairine. It cannot be affirmed with certainty that this residuum is therapeutically efficacious, but just as little can it be denied, and certainly the residuum is not contained in the analyses of the artificial mineral waters. Further, in mineral waters, carbonic acid gas occurs both free and in chemical combination. In 1882 it was discovered that carbonic acid gas forms a hydrate, that is to say, that there exist several kinds of carbonic acid gas. The presence of a carbonic acid gas hydrate had long been concluded from the existence of another combination that is a derivative of it, namely, carbonic acid ethyl.

There is a further reason for thinking that minimal quantities of substances in mineral waters may be of importance. The salt mixture forms a whole from which no part can be taken away without disturbing the equilibrium. This shows the fallacy of the old-fashioned notion that springs, the chief ingredients of which are the same, have the same therapeutic effect even though differing in some minor ingredients. There is no analysis so exact or sensitive as our senses—taste and smell. A perfume of musk in the air is perceived by the smell even though it cannot be demonstrated by chemical analysis. Even the best manufactured artificial waters differ from the natural ones in taste and value. This difference is not easy to explain. It is sometimes found, however, that the two mineral waters, otherwise identical, differ as regards electrical conductibility.

As to the so-called "indifferent" springs it is a mistake to speak of them as of minor value. It must be remembered that they too contain mineral ingredients, if only in minimal quantities, which counteract the harmful properties of perfectly pure distilled water. Even hydropathy is a mineral water treatment, for if the water used were without traces of mineral substances it would be poisonous. This has been sufficiently proved elsewhere.—*British Medical Journal.*

